# Lower respiratory tract infection hospitalizations among American Indian/Alaska Native children and the general United States child population

**DOI:** 10.3402/ijch.v74.29256

**Published:** 2015-11-05

**Authors:** Eric M. Foote, Rosalyn J. Singleton, Robert C. Holman, Sara M. Seeman, Claudia A. Steiner, Michael Bartholomew, Thomas W. Hennessy

**Affiliations:** 1 Department of Pediatrics, University of Washington School of Medicine, Seattle, WA, USA; 2 Division of Community Health Services, Alaska Native Tribal Health Consortium, Anchorage, AK, USA; 3 Arctic Investigations Program, National Center for Emerging and Zoonotic Infectious Diseases (NCEZID), Centers for Disease Control and Prevention (CDC), Anchorage, AK, USA; 4 Division of High-Consequence Pathogens and Pathology, NCEZID, CDC, Atlanta, GA, USA; 5 Healthcare Cost and Utilization Project, Center for Delivery, Organization and Markets, Agency for Healthcare Research and Quality, Rockville, MD, USA; 6 Division of Epidemiology and Disease Prevention, Indian Health Service, Rockville, MD, USA

**Keywords:** pneumonia, epidemiology, American Indian, respiratory, Alaska Native

## Abstract

**Background:**

The lower respiratory tract infection (LRTI)-associated hospitalization rate in American Indian and Alaska Native (AI/AN) children aged <5 years declined during 1998–2008, yet remained 1.6 times higher than the general US child population in 2006–2008.

**Purpose:**

Describe the change in LRTI-associated hospitalization rates for AI/AN children and for the general US child population aged <5 years.

**Methods:**

A retrospective analysis of hospitalizations with discharge ICD-9-CM codes for LRTI for AI/AN children and for the general US child population <5 years during 2009–2011 was conducted using Indian Health Service direct and contract care inpatient data and the Nationwide Inpatient Sample, respectively. We calculated hospitalization rates and made comparisons to previously published 1998–1999 rates prior to pneumococcal conjugate vaccine introduction.

**Results:**

The average annual LRTI-associated hospitalization rate declined from 1998–1999 to 2009–2011 in AI/AN (35%, p<0.01) and the general US child population (19%, SE: 4.5%, p<0.01). The 2009–2011 AI/AN child average annual LRTI-associated hospitalization rate was 20.7 per 1,000, 1.5 times higher than the US child rate (13.7 95% CI: 12.6–14.8). The Alaska (38.9) and Southwest regions (27.3) had the highest rates. The disparity was greatest for infant (<1 year) pneumonia-associated and 2009–2010 H1N1 influenza-associated hospitalizations.

**Conclusions:**

Although the LRTI-associated hospitalization rate declined, the 2009–2011 AI/AN child rate remained higher than the US child rate, especially in the Alaska and Southwest regions. The residual disparity is likely multi-factorial and partly related to household crowding, indoor smoke exposure, lack of piped water and poverty. Implementation of interventions proven to reduce LRTI is needed among AI/AN children.

Lower respiratory tract infection (LRTI) is the leading cause of hospitalization in US children aged <5 years (1). Pneumonia and bronchiolitis are the most common causes of LRTI-associated hospitalizationsin US children aged <5 years (2). Among American Indian and Alaska Native (AI/AN) children, there have been persistently higher rates of LRTI-associated hospitalizations compared to the general US child population (2–4). We last examined this disparity for 2006–2008 and found that the LRTI-associated hospitalization rate for AI/AN children aged <5 years was approximately 1.6 times higher than the corresponding general US child rate, with greater disparities in AI/AN infants (<1 year) and in AI/AN children in Alaska and the Southwest regions (2). Since 2008, the H1N1 outbreak occurred (2009–2010) and the 13-valent pneumococcal conjugate vaccine (PCV13) replaced 7-valent PCV in March 2010 (5). In the present study, we describe the change in LRTI-associated hospitalization rates in AI/AN children and in the general US child population aged <5 years between 1998–1999 and 2009–2011 to compare rates before and after the introduction of the PCV vaccine and the H1N1 influenza outbreak.

## Methods

This manuscript was reviewed and approved for publication by the Alaska Native Tribal Health Consortium, Yukon Kuskokwim (YK) Health Corporation, Maniilaq Association and Indian Health Service (IHS).

We performed an analysis of 1998–2011 hospital discharge data with an LRTI-associated diagnosis among 2 populations: AI/AN children aged <5 years who were hospitalized at an IHS-operated, tribally operated or contract hospital, and the general US child population aged <5 years. All hospital discharge records with an LRTI listed as any one of up to 15 diagnoses were selected from the respective data sets: (a) the IHS Direct and Contract Health Service Inpatient Dataset record for AI/AN children (6,7) and (b) the Nationwide Inpatient Sample (NIS) for the general US child population (8). Hospital stays for newborns (births) were excluded, and the unit of analysis is a hospitalization. The *International Classification of Diseases, Ninth Revision, Clinical Modification* (ICD-9-CM) (9) codes consistent with LRTI were previously defined by Peck et al. as any one of the following diagnoses (ICD-9-CM codes in parentheses): pulmonary tuberculosis (011), pulmonary anthrax (022.1), pulmonary diseases caused by Mycobacterium (031.0), whooping cough (033), respiratory syncytial virus (RSV) (079.6), syphilis of lung (095.1), acute bronchitis (466.0), acute bronchiolitis (466.1), pneumonia (480–486), influenza (487), influenza due to identified 2009 H1N1 influenza virus (488.1), empyema (510), pleurisy with effusion (511.1), abscess of lung and mediastinum (513), rheumatic pneumonia (517.1) and congenital pneumonia (770.0) (2,3). Acute bronchiolitis, pneumonia, RSV (466.11, 480.1 and 079.6) and influenza (487 and 488.1) were further examined. A bronchiolitis-associated hospitalization without pneumonia listed as a diagnosis and percentage of LRTI-associated hospitalizations due to pneumonia were also analyzed. The data were examined by time period, age group (infant and 1–4 years), sex, IHS region (for AI/ANs), LRTI category (bronchiolitis, pneumonia, RSV and influenza) and hospital length of stay.

For AI/AN children aged <5 years, IHS direct and IHS-contract health service inpatient data were obtained from the IHS National Patient Information Reporting System (2,7,10). These data consisted of all hospital discharge records reported directly from IHS- and tribally operated hospitals and community hospitals that are contracted with IHS or tribes to provide health care services to eligible AI/AN people (6). The IHS administrative areas were classified by region: East (Nashville); Northern Plains (Aberdeen, Bemidji and Billings); Alaska, Southern Plains and Southwest (Albuquerque, Navajo, Phoenix and Tucson). We excluded the IHS California and Portland (Oregon) Administrative Areas because neither had any IHS- or tribally operated hospitals (6,11).

The average annual rates per 1,000 AI/AN children were calculated from corresponding groups using the IHS user population which includes all registered AI/AN people who received IHS-funded health care service at least once during the preceding 3 years (2,6). Influenza-associated infant hospitalizations were examined for April 2009 to March 2010 to take into account the influenza season using the corresponding IHS user population for that period. The Alaska IHS region was further analyzed for the YK Delta and Maniilaq sub-regions, which had the highest LRTI-associated average annual hospitalization rates, for 2001–2003 and 2009–2011 (8,12,13).

For the general US child population aged <5 years, LRTI-associated hospitalizations were examined using the NIS, a national representative sample of hospitals conducted by the Healthcare Cost and Utilization Project (HCUP) (8). Participating hospitals are short-term, non-federal general and specialty hospitals sampled annually; these data do not include IHS/tribal facilities’ hospital discharge data. National estimates of the number of hospitalizations were calculated using the HCUP weighting methodology for the NIS (8). SUDAAN software was used to account for the sampling design of the US nationwide inpatient data and generate standard errors (SEs) of hospitalization estimates (14). Average annual hospitalization rates per 1,000 children with 95% confidence intervals (CIs) from 2009 to 2011 were calculated for corresponding groups with denominators determined from the National Center for Health Statistics bridged race population estimates (8,15). Additionally, an annual infant influenza-associated hospitalization rate per 1,000 infants with a 95% CI from April 2009 to March 2010 was calculated.

Overall, average annual rates for 2009–2011 were also compared to corresponding previously published average annual rates for 1998–1999 and 2006–2008 for LRTI, bronchiolitis, pneumonia and RSV; rate ratios (RRs) with 95% CIs were calculated using Poisson regression analysis for IHS data, (16) and relative SEs for ratio estimators were calculated using SUDAAN for NIS data (17). The hospital discharge data used for these earlier time periods were available for the analysis (2,3). The average annual percent rate of change between 1998–1999 and 2009–2011 rates was also calculated. Median length of stay comparison by period was performed using the Wilcoxon rank-sum test. Statistical significance was considered at the p<0.05 level.

## Results

### LRTI-associated hospitalizations

The average annual LRTI-associated hospitalization rate for 2009–2011 in AI/AN children aged <5 years was 20.7 per 1,000, approximately 1.5 times greater than the general US child population aged <5 years (13.7, 95% CI: 12.6–14.8) (Table I and Table II). The average annual LRTI-associated hospitalization rate declined from 1998–1999 to 2009–2011 in both AI/AN (35% decline, p<0.01) and US children (19% decline, SE: 4.5%, p<0.01) (Fig. 2). The 2009–2011 LRTI-associated hospitalization rate was 5% less (p<0.01) than the 2006–2008 rate of 21.8 per 1,000 for AI/AN children, while the rate for US children was not significantly different than the 2006–2008 rate (RR 0.99, 95% CI: 0.88–1.10). For 2009–2011, 50% of all hospitalizations in AI/AN children were LRTI associated compared to 28% (SE: 0.6%) in the general US child population.

**Table I:** Average annual rates of lower respiratory tract infection (LRTI)-, bronchiolitis- and pneumonia-hospitalizations among American Indian and Alaska Native children aged <5 years, by age group and sex, 1998–1999 and 2009–2011^a^

	Lower respiratory tract infection			Pneumonia			Bronchiolitis		
			
Characteristic	1998–1999 rate^b^	2009–2011 rate^b^	Rate ratio (95% CI)^c^	1998–1999 rate^b^	2009–2011 rate^b^	Rate ratio (95% CI)^c^	1998–1999 rate^b^	2009–2011 rate^b^	Rate ratio (95% CI)^c^
Age									
< 1	121.5	75.1	0.62 (0.59–0.64)^d^	55.7	36.9	0.66 (0.62–0.71)^d^	77.3	44.6	0.58 (0.55–0.61)^d^
1–4	15.3	11.3	0.74 (0.70–0.78)^d^	11.4	7.9	0.69 (0.66–0.74)^d^	4.6	4.2	0.91 (0.83–0.99)^d^
Sex by age (year)									
Male	34.1	22.2	0.65 (0.62–0.68)^d^	19.4	12.9	0.67 (0.63–0.71)^d^	17.0	11.0	0.65 (0.61–0.69)^d^
< 1	133.4	82.6	0.62 (0.58–0.66)^d^	60.6	40.3	0.67 (0.61–0.72)^d^	84.8	50.3	0.59 (0.55–0.64)^d^
1–4	15.6	11.8	0.76 (0.74–0.81)^d^	11.7	8.2	0.70 (0.65–0.76)^d^	4.3	4.2	0.97 (0.85–1.09)
Female	29.7	19.1	0.64 (0.61–0.67)^d^	17.2	11.4	0.66 (0.62–0.70)^d^	14.9	9.2	0.62 (0.58–0.66)^d^
< 1	109.0	67.2	0.62 (0.58–0.66)^d^	50.6	33.4	0.66 (0.60–0.72)^d^	69.3	38.8	0.56 (0.51–0.61)^d^
1–4	15.0	10.8	0.72 (0.67–0.78)^d^	11.0	7.6	0.69 (0.63–0.75)^d^	4.9	4.1	0.85 (0.76–0.96)^d^
Region by age (year)									
Alaska	51.8	38.9	0.75 (0.70–0.81)^d^	29.5	22.6	0.77 (0.70–0.84)^d^	28.6	16.3	0.57 (0.51–0.63)^d^
< 1	190.4	136.4	0.72 (0.66–0.78)^d^	92.6	67.6	0.73 (0.64–0.83)^d^	125.1	68.2	0.55 (0.48–0.61)^d^
1–4	21.4	17.0	0.79 (0.70–0.90)^d^	15.7	12.5	0.80 (0.69–0.92)^d^	7.5	4.6	0.62 (0.50–0.77)^d^
East	21.5	10.8	0.50 (0.40–0.63)^d^	15.1	6.1	0.41 (0.30–0.54)^d^	7.2	4.9	0.68 (0.47–0.99)^d^
< 1	66.6	33.3	0.50 (0.35–0.71)^d^	42.6	11.9	0.28 (0.17–0.47)^d^	29.5	22.8	0.77 (0.48–1.25)
1–4	14.0	6.9	0.49 (0.36–0.68)^d^	10.5	5.1	0.49 (0.34–0.70)^d^	3.4	1.8	0.52 (0.28–0.97)^d^
Northern Plains East	5.5	3.5	0.63 (0.47–0.85)^d^	4.0	2.4	0.60 (0.42–0.85)^d^	1.9	1.2	0.66 (0.40–1.10)
< 1	22.5	13.4	0.60 (0.39–0.91)^d^	14.4	9.4	0.65 (0.39–1.09)	10.2	4.9	0.48 (0.25–0.93)^d^
1–4	3.0	2.1	0.72 (0.47–1.09)	2.4	1.4	0.59 (0.36–0.96)^d^	0.6	0.8	1.17 (0.51–2.69)
Northern Plains West	32.5	19.6	0.60 (0.56–0.65)^d^	21.9	13.7	0.63 (0.57–0.69)^d^	11.2	7.5	0.67 (0.59–0.76)^d^
< 1	113.7	72.6	0.64 (0.57–0.71)^d^	64.8	44.3	0.68 (0.59–0.79)^d^	53.7	37.3	0.69 (0.60–0.81)^d^
1–4	16.9	10.7	0.64 (0.56–0.72)^d^	13.6	8.6	0.63 (0.55–0.72)^d^	2.9	2.5	0.84 (0.64–1.09)
Southern Plains	11.9	5.4	0.45 (0.40–0.52)^d^	8.2	2.8	0.34 (0.29–0.40)^d^	4.9	3.3	0.67 (0.56–0.80)^d^
< 1	47.1	22.0	0.47 (0.39–0.55)^d^	26.9	8.0	0.30 (0.23–0.39)^d^	28.3	17.7	0.62 (0.51–0.76)^d^
1–4	6.1	2.9	0.47 (0.39–0.57)^d^	5.1	2.0	0.38 (0.31–0.48)^d^	1.1	1.1	1.02 (0.71–1.48)^d^
Southwest	42.6	27.3	0.64 (0.61–0.67)^d^	22.1	15.4	0.70 (0.66–0.74)^d^	23.6	14.9	0.63 (0.59–0.67)^d^
< 1	157.1	90.1	0.57 (0.54–0.61)^d^	61.4	41.9	0.68 (0.62–0.75)^d^	108.4	59.0	0.54 (0.51–0.58)^d^
1–4	20.5	16.4	0.80 (0.75–0.86)^d^	14.5	10.8	0.75 (0.69–0.81)^d^	7.3	7.2	0.99 (0.89–1.1)
Total	31.9	20.7	0.65 (0.63–0.67)^d^	18.3	12.2	0.66 (0.64–0.69)^d^	16.0	10.1	0.63 (0.60–0.66)^d^

a Data from the IHS National Patient Information Reporting System (7).

b Rate per 1,000 children of corresponding group.

c Rate ratio comparing the 1998–1999 rate with the 2009–2011 rate, with 95% CI.

d Rate ratio is significant at the 0.05 level of significance.

**Table II:** Average annual rates of lower respiratory tract infection (LRTI)-, bronchiolitis- and pneumonia-hospitalizations among the general population of US children aged <5 years, by age group and sex, 1998–1999 and 2009–2011^a^

	Lower respiratory tract infection			Pneumonia			Bronchiolitis		
			
Characteristic	1998–1999 rate 95%CI^b^	2009–2011 rate 95% CI^b^	Rate ratio 95% CI^c^	1998–1999 rate 95% CI^b^	2009–2011 rate 95% CI^b^	Rate ratio 95% CI^c^	1998–1999 rate 95% CI^b^	2009–2011 rate 95% CI^b^	Rate ratio 95% CI^c^
Age									
	49.3	36.3	0.73	18.7	11.7	0.63	31.8	26.1	0.82
<1	45.5–53.1	33.1–39.5	0.65–0.82^d^	17.2–20.1	10.8–12.7	0.56–0.70^d^	29.2–34.5	23.7–28.6	0.72–0.93^d^
	9.1	8.2	0.9	6.8	5.5	0.92	1.9	2.8	1.43
1–4	8.4–9.7	7.5–8.8	0.81–1.0	6.3–7.3	5.1–5.9	0.84–0.99^d^	1.8–2.1	2.5–3.0	1.24–1.61^d^
Sex by age (year)									
	19.3	15.4	0.80	10.1	7.4	0.73	9.0	8.4	0.92
Male	17.9–20.7	14.1–16.6	0.71–0.88^d^	9.4–10.9	6.8–7.9	0.65–0.80^d^	8.3–9.8	7.6–9.1	0.81–1.04
<1	57.0	41.9	0.73	21.3	13.5	0.63	37.0	30.2	0.81
	52.6–61.5	38.2–45.6	0.65–0.82^d^	19.6–23.0	12.4–14.6	0.56–0.70^d^	33.9–40.1	27.3–33.0	0.71–0.92^d^
1–4	10.0	8.8	0.89	7.4	5.8	0.79	2.2	3.0	1.40
	9.3–10.6	8.1–9.5	0.79–0.98^d^	6.9–7.9	5.4–6.3	0.71–0.87^d^	2.0–2.3	2.7–3.3	1.21–1.58^d^
	14.6	11.9	0.82	8.1	6.0	0.74	6.7	6.3	0.96
Female	13.6–15.7	10.9–12.9	0.72–0.91^d^	7.5–8.7	5.5–6.5	0.66–0.82^d^	6.0–7.1	5.7–6.9	0.84–1.08
<1	41.2	30.3	0.74	15.9	9.8	0.62	26.4	21.9	0.83
	38.0–44.3	27.6–33.1	0.65–0.82^d^	14.7–17.1	9.0–10.6	0.55–0.69^d^	24.2–28.6	19.8–24.0	0.72–0.93^d^
1–4	8.1	7.4	0.91	6.2	5.1	0.82	1.7	2.5	1.46
	7.5–8.7	6.8–8.0	0.81–1.0	5.7–6.6	4.7–5.5	0.73–0.91^d^	1.5–1.8	2.2–2.7	1.27–1.66^d^
	17.0	13.7	0.81	9.1	6.7	0.73	7.8	7.4	0.94
Total	15.8–18.2	12.6–14.8	0.72–0.89^d^	8.5–9.8	6.2–7.2	0.66–0.81^d^	7.2–8.5	6.7–8.0	0.82–1.06

a Data from Nationwide Inpatient Sample (NIS) (8).

b Rate per 1,000 children of corresponding group, with 95% CI.

c Rate ratio comparing the 1998–1999 rate with the 2009–2011 rate, with 95% CI.

d Rate ratio is significant at the 0.05 level of significance.

For 2009–2011, the median length of stay for LRTI-associated hospitalizations was 2.0 days (quartiles 1.0, 3.0 days) for AI/AN children and 1.9 days (quartiles 1.1, 3.3 days) for US children. The median length of stay for AI/AN children decreased from 3.0 days in 1998–1999 (quartiles 2.0, 4.0, p<0.01), but did not change for US children (p=0.32).

### Pneumonia-associated hospitalizations

**Fig. 1:**
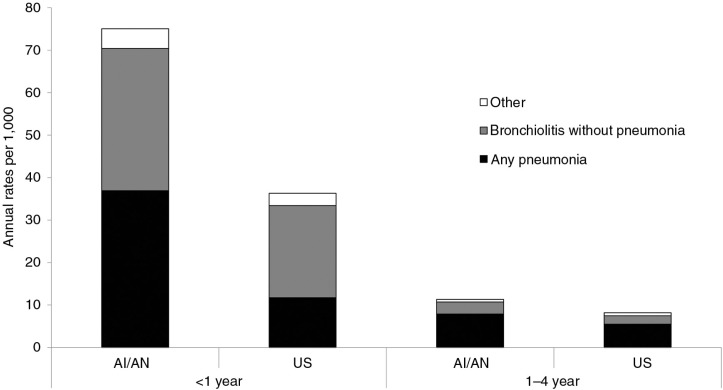
Average annual hospitalization rates associated with lower respiratory tract infection (LRTI) by disease category (any pneumonia, bronchiolitis without pneumonia and other LRTI diagnoses) among American Indian/Alaska Native (AI/AN) children and among the general US child population <5 years of age, by age group, 2009–2011. Data from the IHS National Patient Information Reporting System (7) for AI/AN children and the Nationwide Inpatient Sample for the general US child population (8).

**Fig. 2:**
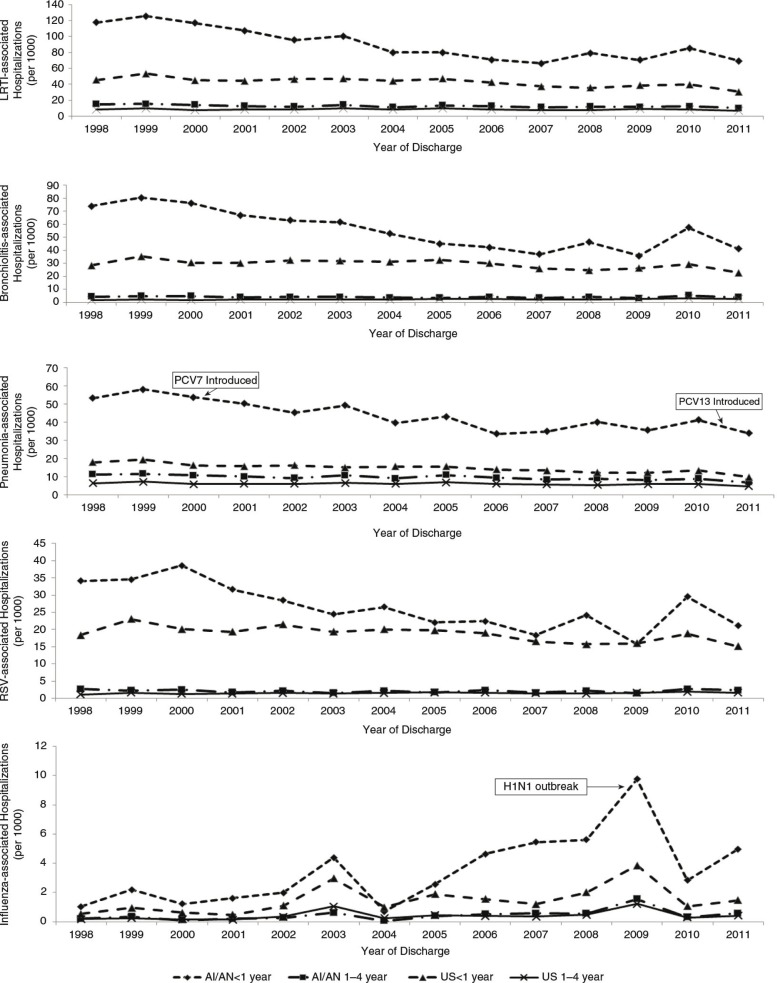
Annual hospitalization rates associated with lower respiratory tract infection (LRTI), bronchiolitis, pneumonia, respiratory syncytial virus (RSV) and influenza among American Indian and Alaska Native (AI/AN) children and among the general US child population <5 years by age group, 1998–2011. Data from IHS National Patient Information Reporting System (7) for AI/AN children and the Nationwide Inpatient Sample for the general US child population (8).

The 2009–2011 average annual pneumonia-associated hospitalization rate for AI/AN children (12.2) was almost 2 times higher than the US child rate (6.7, 95% CI: 6.2–7.2), while the AI/AN infant pneumonia-associated hospitalization rate (36.9) was approximately 3 times higher than the US infant rate (11.7, 95% CI: 10.8–12.7) (Fig. 1, Table I and Table II). The rates declined from 1998–1999 to 2009–2011 for AI/AN infants (34%, p<0.01) and US infants (37%, SE: 3.5%, p<0.01), and continued to decline during 2009–2011 (Fig. 2). AI/AN infants had a greater percentage of LRTI-associated hospitalizations due to pneumonia than US infants (34 and 20%, SE: 0.5%, respectively).

The 2009–2011 average annual pneumonia-associated hospitalization rate for AI/AN infants was highest in Alaska (67.6 per 1,000), and approximately 6 times higher than the general US infant rate (Table I). Within Alaska, the YK Delta sub-region infant hospitalization rate (117.4) was approximately 10 times higher than the US rate, but decreased by 49.4% from 2001 to 2003 (232.2, p<0.01). In contrast, the 2009–2011 infant hospitalization rate in Maniilaq sub-region in Alaska (172.5) was approximately 15 times higher than the US infant rate, and increased by 98.3% from 2001 to 2003 (87.0, p<0.01).

### Bronchiolitis-associated hospitalizations

The 2009–2011 average annual bronchiolitis-associated hospitalization rate for AI/AN infants was approximately 1.7 times higher than that for US infants [44.6 and 26.1 (95% CI: 23.7–28.6), respectively] (Table I and Table II). The rate decreased from 1998–1999 to 2009–2011 for both AI/AN infants (42%, p<0.01) and US infants (18%, SE: 5.3%, p<0.01) (Fig. 2).

### RSV-coded hospitalizations

The 2009–2011 RSV-coded average annual hospitalization rates for AI/AN and US infants were 22.1 and 16.6 (95% CI: 15.1–18.2), respectively (Table I and Table II). Over the period spanning 1998–1999 to 2009–2011, the rate of RSV-related hospitalization declined by 36% for AI/AN infants (p<0.01) and 20% (SE: 5.1%) for US infants (p<0.01) (Fig. 2). Alaska and the Southwest had the highest regional AI/AN infant RSV-associated hospitalization rates (43.1 and 25.4, respectively). Alaska experienced the largest regional infant hospitalization rate decline (52%, p<0.01) from 1998–1999 to 2009–2011 among IHS regions.

### Influenza-associated hospitalizations

For April 2009–March 2010, the 2009 H1N1 influenza pandemic period, the annual rate of infant influenza-associated hospitalizations was approximately 3 times higher for AI/AN infants (9.9) than US infants (3.3, 95% CI: 2.7–2.9) and about 12 times higher for AI/AN infants in Alaska (38.9) (Fig. 2).

## Discussion

The average annual rate of LRTI-associated hospitalizations in AI/AN children and in the general US child population aged <5 years declined significantly from 1998–1999 to 2009–2011. However, there remains a persistent disparity in the rate of LRTI-associated hospitalizations between AI/AN children and the general US child population. The 2009–2011 AI/AN rate was 1.5 times higher than the general US child population rate, which was similar to the disparity from 2006 to 2008 (1.6 times higher) but slightly less than the disparity from 1998 to 1999 (1.9 times higher) (2,3). The disparity in LRTI-associated hospitalization rates in 2009–2011 was greatest for AI/AN infants (2.1 times higher than the general US child population). LRTI-associated hospitalizations accounted for about half of all hospitalizations in AI/AN children compared to about one-quarter of all hospitalizations for US children. The rate disparity was much greater in the IHS Alaska and Southwest regions. Infant pneumonia-associated hospitalization rates in 2 rural Alaska sub-regions were 10–15 times the rates for US infants.

Other studies have reported that AI/AN children have higher rates of morbidity mortality from pneumonia compared to the general US child population. Groom et al. (4) reported a mortality rate for pneumonia and influenza 4.8 times higher for AI/AN infants than US white infants during 1990–2009. Bronchiectasis secondary to childhood pneumonia is a rare lung condition in US children, but it is commonly diagnosed in AI/AN children living in Alaska (14–20 per 1,000 births) similar to Australian and New Zealand Indigenous children (18). Pneumonia with empyema and invasive pneumococcal disease have also been more common among AI/AN children (12,19,20).

Various environmental exposures may in part explain this disparity. Household crowding, indoor smoke, lack of piped water and poverty have been associated with LRTIs and are more common in the Alaska and Southwest regions (21–24). During the study period, approximately 27% of households in rural regions of Alaska did not have access to in-home water service (24). Lack of access to piped water and having less than 2 sinks in the home have been shown to be risk factors for LRTIs in children, factors which could be related to an inability to perform adequate hand hygiene (24,25). Increasing access to piped water reduced rates of LRTI-related clinic encounters in AI/ANs in Alaska (26).

Crowding was more common among AI/AN households; in the 2000 census, household crowding (>1 person per room) occurred in 5.8% of US households overall compared with 19% of households in US Census-designated AI/AN, Hawaiian areas (15), and 39% households in Alaska's YK Delta sub-region (25). Indoor smoke from woodstoves and tobacco smoke was common in AI/AN homes, particularly in the rural Alaska and Southwest regions (23,25,27,28). In the Southwest region, presence of a wood stove increased the odds of LRTI by 4.9 times (27). Passive tobacco smoke exposure occurred in 46–75% of AI/AN households compared to 18% of US households (23,29). Decreasing indoor smoke from cook stoves reduced physician-diagnosed episodes of childhood pneumonia in the RESPIRE study in Guatemala (30).

The decline in pneumonia-associated hospitalization rates is likely in part due to the introduction of the PCV7 in 2000. Griffin et al. (31) estimated that by 2007–2009, the pneumonia-associated hospitalization rate in US children <2 years fell by 43%. We found similar reductions of pneumonia hospitalization rates in AI/AN infants (34%) and children aged 1–4 years (9.9%) from 1998–1999 to 2009–2011; however, our measured reduction did not specifically quantify the impact of PCV7 on pneumonia hospitalizations since our analysis included all listed pneumonia diagnoses, while Griffin et al. (31) limited pneumonia hospitalizations to the first-listed discharge diagnosis or pneumonia with a first-listed diagnosis of meningitis, septicaemia or empyema.

There was a disparity in completion rates of the 4 dose PCV vaccine between AI/AN and the US non-Hispanic white child population noted in 2009 (71% vs. 83%, respectively) and 2011 (75% vs. 85%, respectively); (32,33). However, the 2010 PCV coverage rate was similar for AI/AN children compared to the white US population (32). PCV13 was introduced in March 2010, and coverage increased to >70% of the US birth cohort by June 2011 (5). In one region of Alaska, 91% of AI/AN children aged <5 years had received at least one dose of PCV13 by August 2011 (34). Griffin et al. (35) observed a 27% decline in pneumonia-associated hospitalizations in children aged <2 years after introduction of PCV13. We observed a decrease in AI/AN pneumonia-associated hospitalizations during 2011 compared to 2009–2010 (Fig. 2); however, we could not assess the impact of PCV13 on pneumonia hospitalizations due to limited time since introduction.

Other factors such as changing hospitalization practices, improved socioeconomic conditions, community education and improved household and environmental factors may have contributed to the decline in pneumonia-associated hospitalization rates in AI/AN children, before and after introduction of PCV (2,3). Evaluation of these practices could elucidate the differing trends in rates among Alaska sub-regions.

The bronchiolitis-associated hospitalization rate increased in US infants from 1980 to 1996, thought to be due to more medically complex children, increased oximetry use, increased daycare use and increased RSV virulence (36,37). In the present study, the bronchiolitis-associated hospitalization rate for 2009–2011 decreased since 1998–1999 for both AI/AN and the general US infant population. Another study described a similar decrease for US children aged <2 years during 2000–2009 (38). Possible causes of decreased bronchiolitis-associated hospitalizations could include a decrease in disease incidence, differing physician practices regarding hospitalization, decrease in passive smoke exposure, differing child care practices, improved hygiene practices and PCV (29,38,39).

The AI/AN infants in the Alaska and Southwest regions had the highest RSV-coded hospitalization rates. The RSV ICD-9-CM diagnosis coding may vary based on specimen collection and coding practices; however, during 18 years of RSV surveillance in Alaska's YK Delta RSV testing procedures remained stable and RSV-associated hospitalization was associated with crowding, lack of piped water, prematurity, and other underlying chronic medical conditions and lack of breastfeeding (22,24,40). Exclusive breastfeeding rates remain relatively low in AI/AN infants through 3 months (40%) and 6 months (12%) of age, similar to the national average (37% and 17%, respectively) (41). One study suggested that exclusive breastfeeding for 4 months decreased the odds of a doctor-attended LRTI visit by approximately 50% for children less than 12 months of age (42). During this RSV surveillance period, improvements in piped water and decreased household crowding were associated with declines in RSV- and LRTI-associated hospitalization (43).

During the 2009–2010 H1N1 influenza pandemic, AI/AN persons experienced higher morbidity and mortality than the general US population (43,44). In this analysis, the influenza-associated hospitalization rate was 3 times higher in AI/AN infants than in US infants. In the Alaska region, the AI/AN infant hospitalization rate was approximately 12 times greater. Influenza vaccine coverage remained relatively low in AI/AN children; in 2010–2011, 67% of AI/AN children aged from 6 months to 4 years were vaccinated (45). Although the influenza vaccine coverage rate in AI/AN children was similar to the US general child population, given the high burden of influenza in AI/AN children, increasing coverage could decrease influenza-associated morbidity and mortality (45,46). One report suggested that requiring influenza vaccination in child care and pre-school settings increased vaccination coverage and reduced rates of influenza-associated hospitalizations in children (46). Administering the influenza vaccine to pregnant women was also effective in reducing influenza-associated hospitalizations in infants in both the general US population and in AI/AN populations, yet maternal influenza vaccination rates remain low (47–49).

The present study has some limitations. Diagnoses may be miscoded or incomplete and may vary by hospital and/or area. Hospital admission criteria, ICD-9-CM diagnostic coding, and/or pathogen-specific diagnostic workups for RSV or influenza, and sensitivity or specificity of diagnostic tests may vary between hospitals. The LRTI trends during the H1N1 influenza pandemic are not representative of long-term trends (50). No standard criteria for pneumonia diagnosis are in use nationally, so pneumonia rate comparisons should be made with caution (51,52). The IHS data set only includes hospital discharge data from IHS/tribal and IHS-contract hospitals; records for AI/AN people hospitalized outside of the IHS health care system were not included. The NIS data set may include hospitalizations of AI/AN children who were hospitalized outside of the IHS/tribal health care system (6). The population used to calculate the hospitalization rates for AI/AN children was an estimate of all children using the IHS/tribal health care system, which may not be representative of the total AI/AN childhood population. The number of states reporting to HCUP during 1998–1999 was less than that reporting during 2009–2011; however, the NIS from 1993 onward has a large enough frame to produce reliable national estimates (8).

While LRTI-associated hospitalization rates in AI/AN children decreased from 1998–1999 to 2009–2011, the rate remained persistently higher in AI/AN children compared to the general US child population and varied by IHS region. Analysis of data from laboratory-confirmed invasive pneumococcal disease, RSV and other respiratory viruses in the Alaska and Southwest IHS regions demonstrate similar trends in these endpoints (12,20,53). The disparity of LRTI-associated hospitalizations in AI/AN children is likely multi-factorial and in part related to increased exposure to indoor smoke, household crowding, lack of piped water and poverty. Interventions to address these environmental and socioeconomic factors as well as promoting immunization, handwashing and breastfeeding could help in reducing the disparity.

## What is already known on this topic:

1. Lower respiratory tract infections (LRTIs) are the leading cause of hospitalization in US children.
2. American Indian and Alaska Native children are more likely to suffer higher rates of morbidity and mortality due to LRTIs than the general US child population.
3. The disparity between LRTI-associated hospitalizations of American Indian and Alaska Native children and the general US child population has fallen over time.

## What this study adds:

1. American Indian and Alaska Native children experience higher rates of hospitalization associated with LRTIs than the general US child population <5 years of age, while this disparity has declined over time.
2. The use of the PCV7/PCV13 vaccine is likely associated with reductions in LRTI-associated hospitalization rates in AI/AN children and the general US child population.
3. American Indian and Alaska Native infants suffered higher rates of hospitalization from influenza during the H1N1 epidemic in 2009–2010 compared to the general US infant population.
